# The botanical integrity of wheat products influences the gastric distention and satiety in healthy subjects

**DOI:** 10.1186/1475-2891-7-12

**Published:** 2008-04-27

**Authors:** Joanna Hlebowicz, Sandra Lindstedt, Ola Björgell, Peter Höglund, Lars-Olof Almér, Gassan Darwiche

**Affiliations:** 1 Department of Medicine, University of Lund, Malmö University Hospital, Malmö, Sweden; 2 Department of Cardiothoracic Surgery, Lund University Hospital, Lund, Sweden; 3 Department of Radiology, University of Lund, Malmö University Hospital, Malmö, Sweden; 4 Department of Clinical Pharmacology, University of Lund, Lund University Hospital, Lund, Sweden

## Abstract

**Background:**

Maintenance of the botanical integrity of cereal kernels and the addition of acetic acid (as vinegar) in the product or meal has been shown to lower the postprandial blood glucose and insulin response and to increase satiety. However, the mechanism behind the benefits of acetic acid on blood glucose and satiety is not clear. We hypothesized that the gastric emptying rate could be involved. Thus, the aim of this study was to evaluate the possible influence of maintained botanical integrity of cereals and the presence of acetic acid (vinegar) on gastric emptying rate (GER), postprandial blood glucose and satiety.

**Methods:**

Fifteen healthy subjects were included in a blinded crossover trial, and thirteen of the subjects completed the study. Equicarbohydrate amounts of the following wheat-based meals were studied: white wheat bread, whole-kernel wheat bread or wholemeal wheat bread served with white wine vinegar. The results were compared with a reference meal consisting of white wheat bread without vinegar. The GER was measured with standardized real-time ultrasonography using normal fasting blood glucose <6.1 mmol/l or plasma glucose <7.0 mmol/l as an inclusion criterion. The GER was calculated as the percentage change in the antral cross-sectional area 15 and 90 minutes after ingestion of the various meals. Satiety scores were estimated and blood glucose was measured before and 15, 30, 45, 60, 90 and 120 min after the start of the meal.

**Results:**

The whole-kernel wheat bread with vinegar resulted in significantly higher (<0.05) satiety than the wholemeal wheat bread and white wheat bread with vinegar and the reference bread. Wheat fiber present in the wholemeal wheat bread, or the presence of wheat kernels per se, did not affect the postprandial blood glucose or GER significantly compared with white wheat bread, neither did the addition of vinegar to white bread affect these variables. There was no correlation found between the satiety with antral areas or GER

**Conclusion:**

The present study shows higher satiety after a whole-kernel wheat bread meal with vinegar. This may be explained by increased antral distension after ingestion of intact cereal kernels but, in this study, not by a lower gastric emptying rate or higher postprandial blood glucose response.

**Trial registration:**

NTR1116

## Background

Changing the diet can control the blood glucose level and help prevent the development of type 2 diabetes. The American Diabetes Association recommends a reduced calorie intake and increased intake of dietary fiber and whole grains to prevent the development of type 2 diabetes [1]. Foods with a low glycemic index that are rich in fiber are recommended [1]. The first reported antiglycemic effect of vinegar was by Ebihara and Nakajima [2]. Vinegar decreases the glycemic index of, for example, rice in sushi by about 20–35% [3]. Also, when white vinegar was added to cold storage potatoes as a vinaigrette sauce the glycemic index was lowered in healthy subjects [4]. Vinegar in a salad dressing added to lettuce and ingested with white wheat bread has been shown to reduce the blood glucose response, but the gastric emptying rate (GER) measured by ultrasonography was not delayed [5]. When vinegar was neutralized to pH 6.0 no effects were seen on the postprandial blood glucose response [5]. The decreased postprandial blood glucose response was explained by a mechanism related to acidity and inhibition of digestive amylases [5]. Further, vinaigrette sauce added to a white wheat bread meal has been shown to reduce postprandial blood glucose and insulin responses in healthy subjects; this was explained by delayed gastric emptying, measured indirectly with paracetamol [6]. Insulin sensitivity was improved and postprandial insulin and glucose responses were reduced in insulin-resistant subjects after a meal containing vinegar [7]. However, in healthy subjects, only the postprandial insulin response was reduced, not the blood glucose response, after ingestion of a white bagel with apple cider vinegar [7]. Satiety and eating behavior are complex, but play a key role in energy intake and metabolic control in healthy subjects and in patients with diabetes. Only one previous study on healthy subjects has been conducted to evaluate the effect of vinegar on satiety. That study showed that white wheat bread ingested with vinegar increased and prolonged the feeling of satiety according to a dose-response relation [8].

The term "whole grain" is often used for wholemeal products in which the structure of the cereal grain is destroyed in the flour containing the original dietary fiber, but also for cereal products in which a large proportion of whole cereal grains is intact. However, there seems to be a major difference in metabolic response between whole grain and wholemeal products. The preparation, cooking and particle size of the grain structures may also affect the metabolic response. The glycemic index decreased in patients with type 2 diabetes when increasing proportions of whole grain bulgur (cracked wheat) were substituted for miller flour in bread [9]. However, in another study, the glycemic response did not differ between bulgur and whole wheat kernels in patients with type 1 and type 2 diabetes [10]. This can be explained by the similar particle sizes of bulgur and wheat after chewing. The particle size of wheat has been found to affect the digestion rate and metabolic response in healthy subjects [11]. The wheat germ of the whole grain acts as a natural amylase inhibitor, which can be destroyed during the milling of wheat into wholemeal flour [12].

Only one study has been conducted previously on the effect of whole kernels on gastric emptying, which showed that in healthy subjects the gastric emptying, measured indirectly with paracetamol, was not affected after meals composed of whole-kernel rye bread or wholemeal rye bread compared to with white wheat bread [13]. A high-dietary-fiber meal was found to delay the GER, measured by ultrasonography, in healthy subjects compared with a low-fiber meal [14]. However, another study showed that the GER following a high-fiber meal consisting of whole wheat grain and rye bread, was not different from that following a low-fiber meal [15]. In a previous study using the same wheat bread recipes as in this study and the meals were ingested without vinegar, a significantly lower blood glucose response and higher satiety after whole-kernel wheat bread than after white wheat bread was observed [16]. However, the GER was not evaluated.

These divergent results indicate that not only glucose response, as was previously known, but also satiety and gastric emptying rate seem to be influenced by variables related to processing conditions and botanical structure. The effects of a combination of vinegar and different fiber structures on the postprandial blood glucose response, gastric emptying or satiety has, as far as we know, not been studied previously. Thus, the aim of this study was to evaluate the possible influence of maintaining the botanical integrity of cereals and the addition of acetic acid (as vinegar) on the GER, postprandial blood glucose and satiety. The hypothesis was that products that delay gastric empting rate also lead to higher satiety and decreased postprandial blood glucose.

## Materials and methods

Fifteen healthy subjects were included in the study. However, one male subject was excluded because he was found to have diabetes mellitus, and one female subject was excluded because celiac disease was diagnosed during the study. Thirteen healthy subjects (six men and seven women: mean age 25 ± 4 years [range 22–35 years]; mean BMI 22.8 ± 3.07 kg/m^2 ^[range 17.7–29.7 kg/m^2^]), without symptoms or a prior history of gastrointestinal disease, abdominal surgery or diabetes mellitus, completed the study. The subjects were not receiving any drugs, except two of the women who were taking birth control medication. One subject was a smoker and none was a snuff user. None of the subjects used any drugs on the day of the examination.

White wheat bread was made from 3700 g white wheat flour, 2000 g water and 200 g yeast. The dough was allowed to rise for 20 min at 28°C. The dough was then divided into 440 g pieces and left to rise for a second time, for 35 min at 40°C (RH: 80%). Loaves were baked at 210°C for 22 min with the addition of steam during the first 30 s. The loaves were stored in a freezer at -20°C until used.

The whole-kernel wheat bread was made from 3076 g wheat kernels that were boiled for 20 min in 3076 g water and then cooled at room temperature, after which 1200 g water, 624 g white wheat flour and 200 g yeast were added. The dough was left to rise for 30 min and then divided into 580 g pieces. These pieces were then allowed to rise for a second period of 45 min at 40°C (RH: 80%). Loaves were baked at 200°C for 45 min.

The wholemeal wheat bread was made from 3076 g milled wheat kernels, (500 g of the flour was scaled with 1000 g boiling water) 1200 g water, 624 g white wheat flour and 200 g yeast. The dough was left to rise for 30 min and then divided into 580 g pieces. These were then allowed to rise for a period of 45 min at 40°C (RH: 80%). Loaves were baked at 200°C for 45 min.

The reference and test meals contained 50 g available carbohydrates from bread products. The content of available carbohydrates was analyzed according to Holm et al [17]. The portion size of the white wheat bread was 106.34 g and, besides 50 g available carbohydrates, contained 2.1 g dietary fiber, 1.8 g fat and 8.3 g protein. The portion size of the whole-kernel wheat bread was 132.66 g and contained, besides 50 g available carbohydrates, 7.2 g dietary fiber, 2.9 g fat and 9.2 protein. The portion size of the wholemeal wheat bread was 107.62 g, and contained 7.2 g dietary fiber, 2.9 g fat and 9.2 g proteins, besides the 50 g available carbohydrates. We used the same baking recipes and baking process as Liljeberg et al [16] for the white wheat reference bread and whole-kernel wheat bread; the content of dietary fiber, fat and proteins were thus assumed to be the same as previously described by Liljeberg et al [16]. The wholemeal wheat bread was made from the same recipe as whole-kernel wheat bread but with milled wheat kernels. The three test meals contained one of the three kinds of test bread dipped in 28 g white wine vinegar (5% acetic acid, pH 2.8–3 Druvan, DR Persfood AB, Eslöv, Sweden), which is equivalent to 23 mmol acetic acid in each test meal. Drinking water, 200 ml, was also served. The reference meal contained white wheat bread and water, but without white wine vinegar. The test products and the reference were served in random order during intervals of 1 week. The subjects were examined between 8:00 and 10:00 am after an 8-h fast. Smoking was prohibited for 8 h before and during the test. The fasting blood glucose concentration of each subject was checked on the day of the examination to ensure that it was normal. If the subject reported gastrointestinal symptoms (diarrhea or constipation) on the study day, the examination was postponed. Each meal was ingested within 10 minutes.

The GER was estimated using a previously described standardized ultrasound method [18]. The sonographic examination was performed using two different ultrasound machines (Siemens Acuson Sequoa 512 and Aloka Prof. Sound) with a multi-MHz abdominal transducer. The same machine was used to calculate values of GER. The measurements of the gastric antrum were performed by the same radiologist, who was blinded with regard to the meals. The measurements were made 15 and 90 minutes after the end of meal ingestion (25 and 100 min after the start of the meal). Gastric emptying was expressed as the percentage change of the antral cross-sectional area from 15 to 90 min. Paired t-test was performed before the beginning of the study and power calculations showed a 71% power to detect a 20% change in GER.

Finger-prick capillary samples were collected before and 15, 30, 45, 60, 90 and 120 min after the start of the meal to measure blood glucose levels. Blood glucose concentrations were measured with a HemoCue Glucose system (HemoCue AB, Ängelholm, Sweden). The validated satiety score scale was used according to the method of Haber et al on the basis of a scoring system with grades from -10 (extreme hunger) to 10 (extreme satiety) [19]. Satiety scores were estimated before the meal and 15, 30, 45, 60, 90 and 120 min after the start of the meal, using the same scoring system.

The study was performed according to the Helsinki declaration and was approved by the Ethics Committee at Lund University, and participants provided written informed consent.

The changes from pre-ingestion values in blood glucose and satiety after the different treatments were presented as means ± SEMs and were tested globally in a repeated measures linear mixed model using the interaction of time and treatment as fixed effects and subjects as random effects (SAS, version 8.2, SAS Institute, Cary, NC). For the covariance structure of the repeated measures within a series a spatial exponential model was used. The areas under the curve (AUCs) above zero for delta blood glucose and satiety responses of the four treatments were determined for each subject (Graph Pad PRISM, version 4, San Diego, CA) and presented as means ± SEMs. These were tested globally in a mixed model where treatments were entered as fixed effects and subjects were entered as random effects. Tukey's multiple comparisons test was used as follow-up procedure after the mixed models when appropriate. In addition we tested the inclusion of BMI as covariate in the mixed model for glucose and also the possible correlation between satiety and antral areas or GER. Median values and quartiles are presented for the antral cross-sectional areas and the GER. These were tested globally using the Friedman rank sum test, and when the null hypothesis was rejected, followed by pair-wise comparisons using Wilcoxon rank sum test with the Holm sequential procedure for P-value adjustment (R, version 2.6, The R foundation for statistical computing c/o Institut für Statistik und Wahrscheinlichkeitstheorie, Technische Universität Wien, Wiedner Hauptstraée 8-10/1071,1040 Vienna, Austria). Statistical significance was accepted at p < 0.05.

## Results

### Postprandial blood glucose response

**Figure 1:**
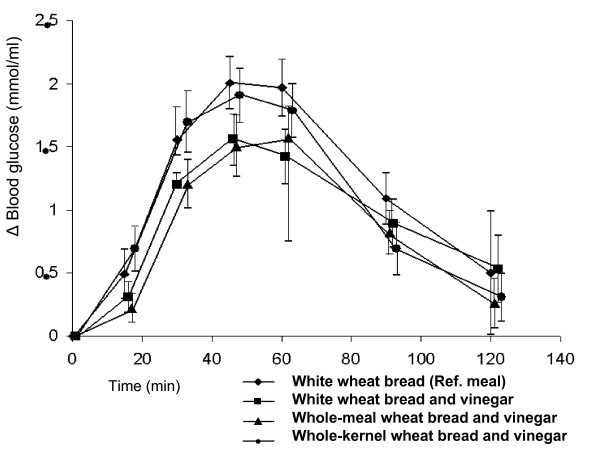
**The mean (± SEM) incremental blood glucose concentration in thirteen healthy subjects after the ingestion of meals consisting of white wheat bread only (reference) (diamond), white wheat bread with vinegar (square), wholemeal wheat bread with vinegar (triangel) and whole-kernel bread with vinegar (dot).** No significant differences were found between the incremental blood glucose concentrations following the various meals.

The mean fasting blood glucose level before the reference meal of white bread was 4.4 ± 0.2 mmol/l compared and not significant different to before ingestion of vinegar together with white wheat bread, wholemeal bread and whole-kernel bread were 4.5 ± 0.1, 4.6 ± 0.1 and 4.5 ± 0.2 mmol min/l, respectively. No significant differences were seen in blood glucose responses at different times, or in the incremental areas under the postprandial glucose curves between the different bread meals (Figure 1). The mean blood glucose AUC 0–120 min after ingestion of the reference meal of white wheat bread was 147 ± 14 mmol min/l. The AUCs after ingestion of vinegar together with white wheat bread, wholemeal bread and whole-kernel bread were 114 ± 12, 110 ± 10 and 135 ± 13 mmol min/l, respectively. The blood glucose AUCs did not differ significantly between the meals, (p = 0.13 in the test of the global hypothesis). The inclusion of BMI as a covariate in the analysis of postprandial blood glucose response did not improve the model.

### Satiety

**Figure 2:**
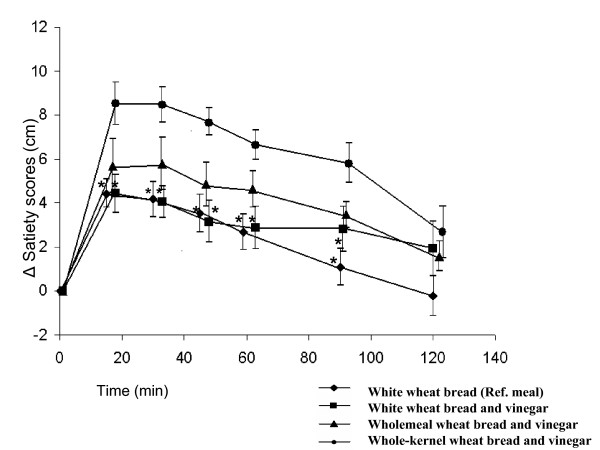
**The mean (± SEM) incremental satiety scores reported by thirteen healthy subjects after the ingestion of meals consisting of white wheat bread (reference) (diamond), white wheat bread with vinegar (square), wholemeal bread with vinegar (triangel), and whole-kernel wheat bread with vinegar (dot).** * Significantly different from the response to whole-kernel bread with vinegar (p < 0.05).

Ingestion of the whole-kernel wheat bread with vinegar resulted in significantly higher satiety scores at 15, 30, 45, 60 and 90 min than the white wheat bread with vinegar and the reference meal, white wheat bread without vinegar (p < 0.05) (Figure 2). Ingestion of whole-kernel wheat bread with vinegar resulted in significantly prolonged satiety, i.e. a higher AUC from 0–120 min, compared with the other bread meals (white wheat bread with vinegar, wholemeal wheat bread with vinegar and the reference white wheat bread, p < 0.05). The mean satiety score after ingestion of the reference meal, i.e. white bread, (AUC from 0–120 min) was 333 ± 56 cm min. The corresponding values after ingestion of the test meals with vinegar were higher: 393 ± 79 cm min for white wheat bread with vinegar, 501 ± 80 cm min for wholemeal bread with vinegar, and 795 ± 82 cm min for whole-kernel wheat bread with vinegar. There was no significant correlation between the satiety with antral areas or GER.

### Gastric emptying rate

**Figure 3:**
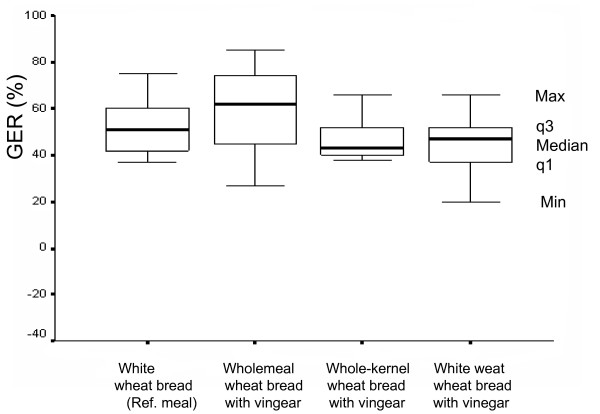
**Gastric emptying rate following the ingestion of white wheat bread with vinegar, wholemeal bread with vinegar, whole-kernel wheat bread with vinegar and white wheat bread (reference), in thirteen healthy subjects.** The median, minimum (Min), and maximum (Max) values and the values of the first (q1) and the third (q3) quartiles are shown. No significant differences were found between the GERs.

No significant differences were observed between the meals with regard to gastric emptying rates (Figure 3). The median value of the GER after the reference meal was estimated to be 51% (q1 = 40%, q3 = 61%) compared with the corresponding value after the reference meal with vinegar, which was estimated to be 47% (q1 = 36%, q3 = 56%). The median value of the GER after the wholemeal wheat bread with vinegar meal was estimated to be 62% (q1 = 39%, q3 = 74%) which can be compared with the median value of the GER after the whole-kernel wheat bread with vinegar meal, of 43% (q1 = 39%, q3 = 53%).

**Figure 4:**
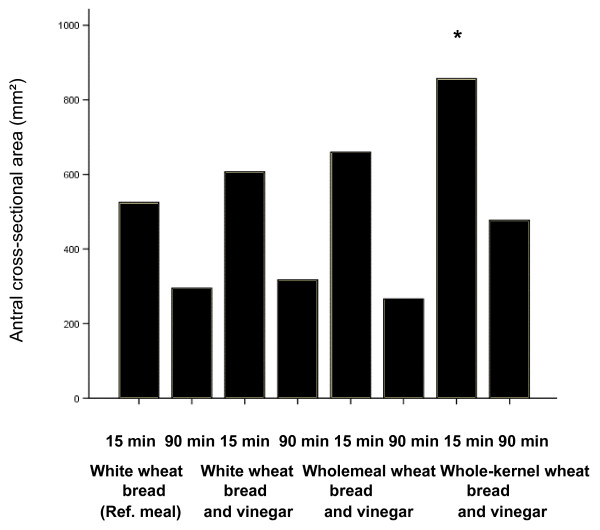
**Median antral area in thirteen healthy subjects 15 and 90 minutes after the end of meal ingestion meals consisting of white wheat bread (reference), white wheat bread with vinegar, wholemeal bread with vinegar, and whole-kernel wheat bread with vinegar.** * Significantly different from the response to the white wheat bread (reference) (p < 0.05).

The median values of the antral cross-sectional area after the ingestion of the reference meal were 525 mm^2 ^(q1 = 431 mm^2^, q3 = 707 mm^2^) and 295 mm^2 ^(q1 = 193 mm^2^, q3 = 364 mm^2^) 15 and 90 min, respectively, after the end of the meal. The median values of the antral cross-sectional area after the ingestion of the reference meal with vinegar were 607 mm^2 ^(q1 = 607 mm^2^, q3 = 1092 mm^2^) and 317 mm^2 ^(q1 = 264 mm^2^, q3 = 507 mm^2^), 15 and 90 min, respectively after the end of the meal. The median values of the antral cross-sectional area after the ingestion of the wholemeal wheat bread with vinegar were 660 mm^2 ^(q1 = 531 mm^2^, q3 = 885 mm^2^) and 266 mm^2 ^(q1 = 166 mm^2^, q3 = 422 mm^2^), respectively, 15 and 90 min after the end of the meal. After the ingestion of the whole-kernel wheat bread with vinegar the median values of the antral cross-sectional area were 857 mm^2 ^(q1 = 657 mm^2^, q3 = 1057 mm^2^) and 477 mm^2 ^(q1 = 329 mm^2^, q3 = 558 mm^2^), respectively, 15 and 90 min after the end of the meal. The median value of the early antral cross-sectional area after the whole-kernel wheat bread with vinegar (857 mm^2^) was significantly larger (p < 0.05 in a pairwise comparison using Wilcoxon rank sum test after the global Friedman ranks sum test being significant p = 0.0022) than the corresponding area after ingestion of the reference meal (525 mm^2^) (Figure 4).

## Discussion

The aim of this study was to elucidate the effect of maintained botanical structure and dietary fiber present in wheat-based bread products in combination with vinegar, on gastric emptying rate, glycemic response and satiety in healthy subjects. Our hypothesis was that an intake of intact cereal kernels with vinegar would increase satiety and lower the postprandial blood glucose response due to delayed gastric emptying. We were not able to verify this hypothesis. The results showed a significant increase in satiety after ingestion of the whole-kernel wheat bread with vinegar compared with the other meals, but no statistically significant differences were seen in gastric emptying rate or postprandial blood glucose response. However, the antral cross-sectional area was significantly larger 15 min after the ingestion of whole-kernel wheat bread with vinegar than after the white wheat reference bread. Thus, the distension of the antrum may explain the increase in satiety scores reported after the whole-kernel wheat bread meal with vinegar compared to the other bread meals with vinegar. However, the antral cross-sectional area did not correlate to the satiety scores. Clearly, a larger trial involving a greater number of subjects would be needed to validate the findings of this small study. A limitation of this study is that the sample size was small. The effects of whole-kernal bread without vinegar were not investigated. However, the white wheat reference bread served with vinegar and compared to white wheat bread without vinegar did not affect the satiety. In a previous study using the same bread recipes but without vinegar, a significantly increased satiety after whole-kernel wheat bread than after white wheat bread was observed [16]. Therefore, it can be assumed that the whole-kernel bread was more satiating than the other meals regardless of adding vinegar. This study thus shows that the botanical structure rather than the amount of fiber per se causes the distension of the antrum and increased satiety. This relationship between antral area and satiety in healthy subjects has been observed previously by others [20-24]. Holt et al have also reported an association between the particle size of wheat and satiety [25].

Another intention of the current study was to evaluate the effect of whole kernels on blood glucose response and gastric emptying. This lack of difference in postprandial blood glucose response between the bread meals was most unexpected as, in a previous study using the same bread recipes but without vinegar, it was observed a significantly lower blood glucose response after whole-kernel wheat bread than after white wheat bread [16]. The botanical integrity of the grain kernels may have been unintentionally destroyed during the baking process, which would explain the present observations. However, the structure of the bread was not investigated. The lack of difference in GER between the bread meals is in agreement with studied performed by Juntunen et al, who compared whole-kernel rye bread and wholemeal rye bread to white wheat bread, despite the known difference in insulin response between rye and wheat [13]. Unfortunately, we did not control the subjects for exercise or food choice the night before of the testing. This may have affected the postprandial blood glucose responses.

Another intention of the current study was to evaluate the effect of vinegar on blood glucose response and gastric emptying. However, we did not observe the lowering effect of vinegar on blood glucose response reported with white wheat bread [8] and potatoes [4] in previous studies. However, when 20 g apple cider vinegar was ingested prior to a low-glycemic meal the postprandial insulin response was lower, but no effect was observed on the blood glucose response in healthy subjects [26]. However, ingestion of 20 g apple cider vinegar prior to a high-glycemic meal composed of bagel, butter and orange juice, reduced the postprandial blood glucose and insulin response in healthy subjects [26]. In a recent study, the postprandial glucose and insulin responses of type 2 diabetes patients were found not to be affected, and in healthy subjects the postprandial blood glucose levels were not affected, but the insulin levels were reduced when apple cider vinegar was consumed prior to the meal [27]. However, it was demonstrated in insulin-resistant subjects that the postprandial insulin and glucose responses were reduced after drinking 20 g apple cider vinegar prior to a meal consisting of white bagel, butter and orange juice [27].

Our findings, that there was no difference in gastric emptying rate after a meal including vinegar, agree with those of Brighenti et al, who found no difference regarding gastric emptying, measured by ultrasonography, after a meal with 20 ml white vinegar (16 mmol acetic acid), although the blood glucose response was reduced in healthy subjects [5]. They explained the lower postprandial blood glucose response as being due to a mechanism related to acidity and the inhibition of digestive amylases. Another study showed that an addition of 20 g white vinegar (18 mmol acetic acid) to a bread meal lowered the postprandial blood glucose and insulin responses in healthy subject, and this was explained by delayed gastric emptying [6]. However, gastric emptying was measured indirectly using paracetamol, which is a less reliable method.

## Conclusion

The present study shows that the post-prandial ratings of satiety were higher after whole-kernel wheat bread meal served with vinegar. This may be explained by increased antral distension after the ingestion of intact cereal kernels.

## Competing interests

The authors declare that they have no competing interests.

## Authors' contributions

JH participated in the design of the study, recruited the subjects, collected the data, performed the statistical calculations and drafted the manuscript. PH performed the statistical calculations and participated in drafting the manuscript. SL participated in drafting the manuscript. GD participated in the design of the study and participated in drafting the manuscript. OB participated in the design of the study and performed the ultrasound examinations. LOA participated in the design of the study and in drafting the manuscript. All authors read and approved the final manuscript. All authors lack any conflict of interest.
